# Preoperative detection of KRAS G12D mutation in ctDNA is a powerful predictor for early recurrence of resectable PDAC patients

**DOI:** 10.1038/s41416-019-0704-2

**Published:** 2020-01-23

**Authors:** Shiwei Guo, Xiaohan Shi, Jing Shen, Suizhi Gao, Huan Wang, Shuo Shen, Yaqi Pan, Bo Li, Xiongfei Xu, Zhuo Shao, Gang Jin

**Affiliations:** Department of Hepatobiliary Pancreatic Surgery, Changhai Hospital, Navy Military Medical University (Second Military Medical University), Shanghai, China

**Keywords:** Pancreatic cancer, Tumour biomarkers, Predictive markers, Prognostic markers, Pancreatic cancer

## Abstract

**Background:**

About 25–37% of resectable pancreatic ductal adenocarcinoma (PDAC) had a great chance of early recurrence after radical resection, which is mainly due to preoperative micrometastasis. We herein demonstrated the profiles of ctDNA in resectable PDAC and use of ctDNA to identify patients with potential micrometastasis.

**Methods:**

A total of 113 and 44 resectable PDACs were enrolled in discovery and validation cohorts, separately. A panel containing 50 genes was used to screen ctDNA by an NGS-based assessment with high specificity.

**Results:**

In the discovery cohort, the overall detection rate was 38.05% (43/113). Among positive ctDNA, KRAS mutation had the highest detection rate (23.01%, 26/113), while the others were <5%. Survival analysis showed that plasma KRAS mutations, especially KRAS G12D mutation, had significant association with OS and RFS of resectable PDAC. Plasma KRAS G12D mutation showed a strong correlation with early distant metastasis. In the validation cohort, survival analysis showed similar association between plasma KRAS G12D mutation and poor outcomes.

**Conclusions:**

This study demonstrated that plasma KRAS mutations, especially KRAS G12D mutation, served as a useful predictive biomarker for prognosis of resectable PDAC. More importantly, due to high correlation with micrometastasis, preoperative detection of plasma KRAS G12D mutation helps in optimising surgical selection of resectable PDAC.

## Background

Pancreatic ductal adenocarcinoma (PDAC) is one of the most aggressive malignancies with a 5-year survival rate of <8%, and this is mainly due to late diagnosis and rapid progression.^1^ The majority, i.e., up to 80% of the patients have lost the chance of surgery at the time of diagnosis.^2^ Even for patients with resectable PDAC, a significant portion of them (25–37%) had great chances of early recurrence after undergoing radical resection.^3,4^ The presence of micrometastasis in patients that cannot be detected by current preoperative imaging techniques is the main reason for this quick relapse and as to why such patients cannot benefit from surgery.^5,6^ So, new-generation biomarkers are urgently needed to identify patients with micrometastasis, avoiding unnecessary surgical interventions.

Liquid biopsy biomarkers are gaining interest in cancer research due to their advantages of convenient and non-invasive nature.^7^ Exosomes play an important role in PDAC progression and can be used for early detection, especially Glypican-1-positive exosomes can identify patients with late-stage PDAC and distinguish from patients with precancerous pancreatic lesions.^8^ The value of circulating tumour cells (CTCs) in predicting prognosis has been broadly studied. The US Food and Drug Administration (FDA) has approved the use of CTC as a biomarker for prognostic evaluation in three types of cancers.^9–11^ Cell-free circulating tumour DNA (ctDNA) can be effectively distinguished from normal cell-free circulating DNA (cfDNA) by specific cancer-related mutations.^12,13^ Several studies have proved a high detection rate and potential prognostic value of ctDNA in PDAC.^14–17^ However, the data regarding the complexity of exosome isolation invalidated it in other centres, and heterogeneity as well as rarity of CTCs and ctDNA restrained the application mainly in advanced patients.^18^ Therefore, more stable methods and more validations are necessary before clinical application, especially for patients with early-stage PDAC who are commonly considered as resectable.

In recent years, different procedures for plasma ctDNA processing, library construction and analytical methods have led to incomparable results and confusion in clinical application.^19^ For NGS techniques, random errors usually happened during the exponential amplification process, leading to high false-positive rate, and this has been one of the most important defects.^20^ However, some high-fidelity linear amplification sequencing methods and optimised analytical methods may help to improve.^21^ Hence, in this study, a highly specific ctDNA detection method was used to explore the clinical value of hotspot mutations in resectable PDAC patients and hoped that a new ctDNA biomarker can be used to optimise the surgical selection of PDAC patients and eventually improve the survival time of PDAC.

## Methods

### Patients

Patients with resectable PDAC were collected from Changhai prospective database (Changhai Hospital, Shanghai, China). All patients underwent curative surgery for tumour resection and were histologically confirmed with PDAC. Patients who died of surgical complications within 1 month after surgery have been excluded. All patients provided written informed consent to use their clinical data. The study was conducted in accordance with the national guidelines, and acquired the approval of the Ethics Committee at Changhai Hospital.

### Clinical samples

Approximately 0.5–1 cm^3^ tumour tissue was removed from the centre of the resected lesions, and then was rapidly maintained in liquid nitrogen until DNA extraction. In total, 10 mL of preoperative venous blood was collected from patients in ethylenediaminetetraacetic acid tubes (Vacutainer blood collection tubes, Becton, Dickinson and Company, NJ, USA). Within 2 h of collection, the blood was centrifuged at 1600*g* for 10 min at 4 °C to obtain plasma and a buffy coat layer (containing WBCs). The plasma was then further centrifuged at 16,000*g* for 10 min at 4 °C to pellet any remaining cells. Both plasma and the buffy coat layer were collected and stored at −80 °C. Patients were neither provided with the results of ctDNA sequencing nor any treatment decisions made based on ctDNA results.

### Tumour tissue, white blood cell genomic DNA and ctDNA extraction

Before tumour tissue DNA extraction, at least one frozen resection of each sample was assessed by two specialist pathologists to confirm the tumour cellularity ≥20% (Supplementary Fig. 1). Tumour tissue genomic DNA was extracted from the freshly frozen mass using QIAamp DNA mini kit (Qiagen, Venlo, The Netherlands). WBC genomic DNA was extracted by using the QIAamp DNA blood maxi kit (Qiagen, Venlo, The Netherlands). ctDNA was extracted from 3 to 5 mL of plasma using the QIAamp circulating nucleic acid kit (Qiagen, Venlo, The Netherlands). DNA was extracted according to the manufacturer’s instructions, quantified by a Qubit fluorometer (Life Technologies, Carlsbad, CA, USA), and maintained at −80 °C until subsequent use.

### Next-generation sequencing (NGS) analysis in tumour tissue and white blood cell

The genomic NGS library of tumour tissue and WBC was constructed by using KAPA sequencing library construction kit (Kapa Biosystems, Boston, MA, USA). A panel consisting of all exons from 50 genes that are associated with pancreatic cancer (Supplementary Table 1^22^) was used for capturing. The captured products were purified and sequenced on an Illumina Hi-Seq 2500 (Illumina, San Diego, CA, USA). The average coverage depth for all probes of NGS library was at least 500×. Sequencing results were aligned according to the human reference sequence hg19/GRCh37, and the background noise introduced by random NGS error was removed. The true variants were then identified, and the allele frequency was calculated by comparing the number of unique reads containing a variant to the total number of sequencing reads that mapped according to the position of the variant. Germline variants were identified from WBC genomic DNA sequencing, and were removed from tumour genomic DNA total variants to determine somatic mutations.

### Firefly next-generation sequencing analysis in ctDNA

NGS-based assessment of ctDNA was performed using the Firefly platform (AccuraGen, Inc., Shanghai, China). ctDNA was circularised using CircLigase II single-strand DNA ligase (EpiCentre, Madison, WI, USA) with 10–30 ng of DNA per 20 μL of reaction. After ligation, the samples were treated with exonuclease (NEB, Ipswich, MA, USA) to remove the uncircularised DNA. The circularised DNA was then amplified in a rolling circle amplification reaction using Phi29 DNA polymerase (NEB, Ipswich, MA, USA), and the exonuclease-resistant random primers by using the manufacturer's instructions with slight modifications. The amplification product was then purified by AMPure beads (Beckman Coulter, Brea, CA, USA) and sonicated to short fragments that are suitable for NGS library 7/20 construction by using a Covaris sonicator. NGS sequencing libraries were generated from 100 ng of amplified ctDNA using the KAPA sequencing library construction kit (Kapa Biosystems, Boston, MA, USA) according to the manufacturer’s instructions. After library construction, a panel consisting of all exons from 50 genes associated with pancreatic cancer was used for capturing. The captured products were then purified, sequenced on an Illumina Hi-Seq 2500 (Illumina, San Diego, CA, USA) and the unique sequencing reads were determined by using an AccuraGen proprietary algorithm. The average coverage depth for all probes of Firefly NGS library was approximately 7000×. The sequencing results were aligned to the human reference sequence hg19/GRCh37, and the background noise introduced by random NGS error was removed by AccuraGen proprietary algorithms. The true variants were then identified, and the allele frequency was calculated by comparing the number of unique reads containing a variant to the total number of sequencing reads that mapped according to the position of the variant. Germline variants were identified from WBC genomic DNA sequencing and were removed from ctDNA total variants to determine somatic mutations.

### Droplet digital polymerase chain reaction (ddPCR)

The QX200 system (Bio-Rad, Hercules, CA, USA) was utilised for ddPCR experiments. Multiplex KRAS products for ddPCR were utilised, which included six KRAS mutations (KRAS G12D/G12V/G12R/G12C/G12A/G12S). For conducting the experiments, a reaction volume of 20 µl consisting of cfDNA ranging 4–80 ng (8 µL of cfDNA input in volume) was used. Emulsified PCR reactions were run on a 96-well plate on a MJ thermal cycler (Bio-Rad, Hercules, CA, USA) following the manufacturer’s instructions. Plates were read on a Bio-Rad QX200 droplet reader using QuantaSoft version 1.4.0 software (Bio-Rad, Hercules, CA, USA) to assess the number of droplets that was positive for wild-type KRAS and/or KRAS mutations. A sample without plasma ctDNA was used as a negative control. Three positive events were regularly used as a threshold for false positivity.

### Clinicopathological characteristics

The preoperative clinical variables included age, sex, smoking history, drinking history, status of diabetes mellitus, the first clinical symptom (abdominal pain and jaundice), carcinoembryonic antigen (CEA), carbohydrate antigen 19-9 (CA19-9), surgical procedure and radiologic tumour size. Postoperative variables included the eighth edition of American Joint Committee on Cancer (AJCC) TNM classification, differentiation degree, perineural invasion, pathological margin status, nodal involvement and treatment with adjuvant chemotherapy. Overall survival (OS) was defined as the interval from the date of surgery till the date of patient death or the last follow-up visit after 1 month post surgery. Recurrence-free survival (RFS) was defined as the time interval from the date of surgery till the date of local or regional recurrence, distant metastases, death or the last follow-up visit after 1 month post surgery. The status of recurrence was recorded as no, local, distant and both.

### Statistical analysis

Discrete variables were presented as number and/or percentage, whereas the continuous variables were presented as median and/or mean. Fisher’s exact test was used to report variable association of plasma KRAS mutations. Spearman’s rank correlation coefficient was used to determine the association between ddPCR and Firefly NGS in plasma KRAS G12D allele frequency (AF). Univariate and multivariate Cox regression analysis was used to assess the impact of prognostic factors on OS and RFS. *P*-values less than 0.05 were considered to be statistically significant. Statistical analysis was performed by using SPSS 22.0 for Windows (SPSS, Chicago, IL, USA). Curves for OS and RFS were generated using the R statistical package v.3.5.1 (http://www.r-project.org/), and figures were created using GraphPad Prism (version 7.0; GraphPad, San Diego, CA).

## Results

### Discovery cohort

#### Clinicopathological characteristics of patients

**Fig. 1:**
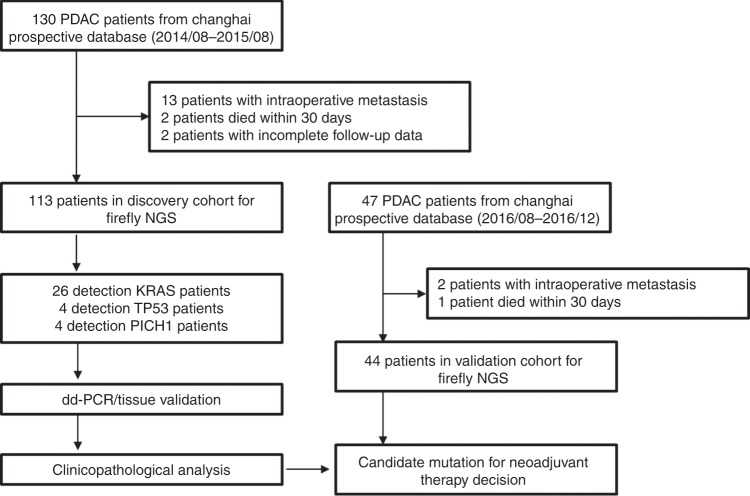
**Workflow diagram of the study.**

From August 2014 to August 2015, a total of 130 consecutive patients diagnosed with resectable PDAC who underwent curative pancreatectomy were enrolled from the Changhai prospective database (Fig. 1). Among these, 13 were found with metastasis during operation, 2 patients died due to surgical complications and 2 patients failed to follow up within 1 month after surgery, and so were excluded from the study. The remaining 113 patients with resectable PDAC were enrolled in this study. Clinicopathological characteristics of 113 patients are summarised in Table 1. The patient cohort enrolled had a median age of 61.5 years, and consisted of a little disproportionately male (69, 61.1%). Patients with stages IA, IB, IA, IIB and III (AJCC, 8th edition) accounted for 15 (13.3%), 34 (30.0%), 14 (12.4%), 43 (38.1%) and 7 (6.2%), respectively. All patients received adjuvant chemotherapy, except six patients due to physical conditions or refusal. Median follow-up of the discovery cohort was 23.6 months.

**Table 1:** Clinicopathological characteristics of PDAC patients enrolled.

Variable	Discovery cohort (*N* = 113)	Validation cohort (*N* = 44)
*Sex*		
Female (%)	44 (38.9%)	15 (34.1%)
Male (%)	69 (61.1%)	29 (65.9%)
*Age at surgery, y*		
Median	61.5	64.5
Range	40–89	48–81
≥70 (%)	24 (21.2%)	10 (22.7%)
*Smoking history*		
No (%)	100 (88.5%)	32 (72.7%)
Yes (%)	13 (11.5%)	12 (27.3%)
*Drinking history*		
No (%)	103 (91.2%)	38 (86.4%)
Yes (%)	10 (8.8%)	6 (13.6%)
*Diabetes mellitus*		
No (%)	80 (70.8%)	29 (65.9%)
Yes (%)	33 (29.2%)	15 (34.1%)
*First clinical symptom*		
Abdominal pain (%)	58 (51.3%)	24 (54.5%)
Jaundice (%)	50 (44.2%)	9 (20.5%)
*CEA at diagnosis, ng/mL*		
Median	3.3	4
Range	0.58–209.25	0.68–43.55
Normal (%)	81 (71.7%)	28 (63.6%)
Elevated (%)	32 (28.3%)	16 (36.4%)
*CA19-9 at diagnosis, U/mL*		
Median	131.4	126.1
Range	<2–>1200	<2–>1200
Normal (%)	22 (19.5%)	12 (27.3%)
Elevated (%)	91 (80.5%)	32 (72.7%)
*Pancreatectomy type*		
Distal pancreatectomy (%)	36 (31.9%)	16 (36.4%)
Pancreaticoduodenectomy (%)	77 (68.1%)	28 (63.6%)
*Radiologic tumour size, cm*		
Median	2.7	3.1
Range	0.2–11.9	0.6–7.5
≤2 (%)	27 (23.9%)	8 (18.2%)
>2, ≤4 (%)	67 (69.3%)	26 (59.1%)
>4 (%)	19 (16.8%)	10 (22.7%)
*Differentiation degree*		
Poor (%)	36 (31.9%)	9 (20.5%)
Medium/well (%)	77 (68.1%)	35 (79.5%)
*Perineural invasion*		
No (%)	23 (20.4%)	2 (4.5%)
Yes (%)	90 (79.6%)	42 (95.5%)
*Pathological margin status*		
R0 (%)	88 (77.9%)	35 (79.5%)
R1 (%)	25 (22.1%)	9 (20.5%)
*Nodal involvement*		
0 (%)	64 (56.6%)	32 (72.7%)
≥1, ≤3 (%)	43 (38.1%)	12 (27.3%)
≥4 (%)	6 (5.3%)	0 (0.0%)
*Pathological stage (AJCC, 8th edition)*		
IA (%)	15 (13.3%)	6 (13.6%)
IB (%)	34 (30.0%)	17 (38.6%)
IIA (%)	14 (12.4%)	9 (20.5%)
IIB (%)	43 (38.1%)	12 (27.3%)
III (%)	7 (6.2%)	0 (0.0%)
*Adjuvant chemotherapy*		
No (%)	6 (5.3%)	3 (7.3%)
Yes (%)	107 (94.7%)	41 (93.2%)
*Recurrent disease*		
No (%)	9 (8.0%)	6 (13.6%)
Yes (%)	104 (92.0%)	38 (86.4%)
*Recurrence types*		
Local (% of total and % of all recurrences)	37 (32.7% and 35.6%)	13 (29.6% and 34.2%)
Distant (% of total and % of all recurrences)	58 (51.3% and 55.8%)	21 (47.7% and 55.3%)
Both (% of total and % of all recurrences)	9 (8.0% and 8.6%)	4 (9.1% and 10.5%)
*ctDNA detection in plasma*		
KRAS (%)	26 (23%)	9 (20.5%)
TP53 (%)	4 (3.5%)	2 (4.5%)
PTCH1 (%)	4 (3.5%)	2 (4.5%)
ROS1 (%)	4 (3.5%)	1 (2.3%)
KIT (%)	3 (2.7%)	2 (4.5%)
MET (%)	3 (2.7%)	0 (0.0%)
BRAF (%)	2 (1.8%)	1 (2.3%)
CDKN2A (%)	2 (1.8%)	1 (2.3%)
SMAD4 (%)	2 (1.8%)	1 (2.3%)
*KRAS mutations in ctDNA*		
KRAS G12D (%)	13 (11.5%)	5 (11.4%)
KRAS G12V (%)	7 (6.2%)	3 (6.8%)
KRAS G12R (%)	2 (1.7%)	1 (2.3%)
KRAS G12C (%)	1 (0.9%)	1 (2.3%)
KRAS G13D (%)	1 (0.9%)	0 (0.0%)
KRAS Q61H (%)	1 (0.9%)	1 (2.3%)
KRAS Q61R (%)	1 (0.9%)	0 (0.0%)

*PDAC* pancreatic ductal adenocarcinoma, *CEA* carcinoembryonic antigen, *CA19-9* carbohydrate antigen 19-9, *AJCC* American Joint Committee on Cancer, *ctDNA* cell-free circulating tumour DNA

#### Detection of pancreatic research panel in ctDNA by Firefly NGS

The Firefly NGS technique allowed a detection of 0.02% mutated DNA.^21^ But according to the previous studies,^18^ we chose 0.1% AF as the threshold to avoid situations, like ageing, benign tumours and preneoplastic lesions, better reflecting the systematic tumour burden of the patients.

Thirty-three genes of the pancreatic research panel were found in the plasma of 43 patients, with an overall detection rate of 38.05% (43/113). Interestingly, except for KRAS, the other genes detected commonly had a pretty low positive rate of <5%. Plasma KRAS mutations were detected in 26 patients, while 87 patients did not have KRAS mutations in their plasma (Table 1). Among the 26 patients with KRAS mutations in ctDNA, KRAS G12D mutation was detected in 13 patients, KRAS G12V in 7 and KRAS G12R in 2. The remaining four patients were detected with KRAS G12C, KRAS G13D, KRAS Q61H and KRAS Q61R, separately.

#### Strong correlation between Firefly NGS and ddPCR in detection of ctDNA

Twenty-six KRAS-positive samples were screened again using multiplex ddPCR (KRAS G12D/G12V/G12R/G12C/G12A/G12S). We compared the AF of KRAS mutation between ddPCR and Firefly NGS. The results showed that the detection of plasma KRAS mutations by ddPCR and Firefly NGS technique was largely equivalent (Supplementary Table 2). For ddPCR-negative subgroup, no false positives were identified by Firefly NGS, suggesting 100% specificity for NGS-based KRAS detection. For ddPCR-positive group, the AF between ddPCR and Firefly NGS was highly concordant (*R*^2^ = 0.98, Supplementary Fig. 2). Moreover, Firefly NGS has successfully profiled three special mutations that were not included in the design of ddPCR (filled in blue, Supplementary Table 2), assisting the previously reported disadvantage of ddPCR for the requirement of detailed prior information on the mutational spectrum.^23^ These results demonstrated that NGS-based platform had similar detection accuracy with that of ddPCR.

#### Validation of plasma KRAS mutation by tissue NGS

Tumour tissues of 26 patients above underwent genomic DNA sequencing using pancreatic research panel. Surprisingly, the results showed that there was one patient who was detected with KRAS G12D mutation in the plasma turned out to be negative in the tissue (filled in green, Supplementary Table 3). This might be due to the spatial heterogeneity within a tumour, which is an advantage of ctDNA^24,25^ or the direct sequencing of surgical sample without microdissection. However, the most important point is that, except for this one, the remaining patients with detected plasma KRAS mutation by Firefly NGS were proven positive in tissue, and consistent in the mutation subtype (Supplementary Table 3). These results demonstrated high specificity of Firefly NGS platform.

#### Patient characteristics associated with plasma KRAS mutation status

A total of 113 patients were divided into two groups according to the presence of KRAS mutations in ctDNA. Clinicopathological characteristics of the two groups are shown in Supplementary Table 4. Most of the patients with KRAS mutation in ctDNA had a history of smoking and drinking, but showed no significant differences between these two groups. Besides, among the other clinicopathological variables included, no significant difference was observed.

#### Significant correlation between plasma KRAS mutations and patient outcomes

**Fig. 2:**
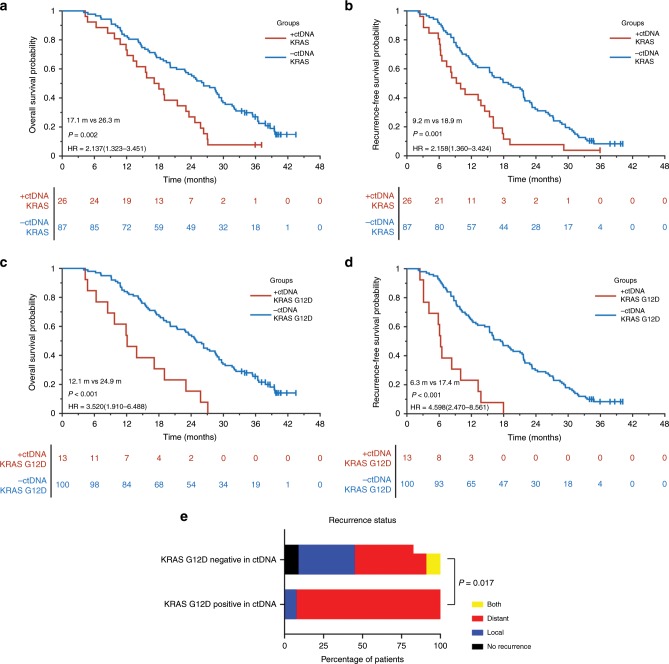
Survival analysis in resectable PDAC patients with or without plasma KRAS and KRAS G12D mutation in the discovery cohort. **a** OS for resectable PDAC patients with (*n* = 26, red) and without (*n* = 87, blue) KRAS mutants in ctDNA. In total, 73.1% of patients with KRAS mutations survived at 12 months post surgery vs 82.8% for the patients without KRAS mutations. **b** RFS for resectable PDAC patients with (*n* = 26, red) and without (*n* = 87, blue) KRAS mutations in ctDNA. In all, 42.3% of patients with KRAS mutations were recurrence-free at 12 months post surgery vs 65.5% for the patients without KRAS mutations. **c** OS for resectable PDAC patients with (*n* = 13, red) and without (*n* = 100, blue) KRAS G12D mutant in ctDNA. **d** RFS for resectable PDAC patients with (*n* = 13, red) and without (*n* = 100, blue) KRAS G12D mutation in ctDNA. **e** Comparison for recurrence types of resectable PDAC patients with (*n* = 13) and without (*n* = 100) KRAS G12D mutation in ctDNA.

Next, the association between KRAS mutations in ctDNA and outcomes of patients was explored. The results showed significant differences in OS (17.1 vs 26.3 months, *P* = 0.002) and RFS (9.2 vs 18.9 months, *P* = 0.001) among patients with detectable KRAS mutations in ctDNA (*n* = 26) when compared with those without KRAS mutations (*n* = 87) (Fig. 2a, b). At 12 months post surgery, the OS was 73.1% (19/26) and 82.8% (72/87) in plasma KRAS mutation positive and negative patients, respectively. The corresponding RFS was 42.3% (11/26) and 65.5% (57/87), respectively.

Among 26 patients with KRAS mutations in ctDNA, patients with plasma KRAS G12D mutation (*n* = 13) showed a significantly reduced OS (12.1 vs 24.9 months, *P* < 0.001) and RFS (6.3 vs 17.4 months, *P* < 0.001) relative to those without plasma KRAS G12D mutations (*n* = 100) (Fig. 2c, d). No such association was found among patients with other assayed KRAS mutations (*n* = 13) (Supplementary Fig. 3A, B).

#### Univariate and multivariate analysis of clinicopathological characteristics for patient outcomes

After that, whether the following patient characteristics were risk factors for OS and RFS were investigated: age, sex, smoking history, drinking history, the first clinical symptom (abdominal pain and jaundice), diabetes mellitus, surgery procedure, CEA, CA19-9, radiologic tumour size, pathological stage (AJCC, 8th edition), differentiation degree, perineural invasion, pathological margin status, nodal involvement and the use of adjuvant chemotherapy. Univariate analysis demonstrated radiologic tumour size, pathological stage (AJCC, 8th edition), nodal involvement, KRAS mutations and KRAS G12D mutation in ctDNA as considerable risk factors for RFS and OS (Table 2). Further multivariate analysis, in which nodal involvement was excluded due to representation in a more general variable (pathological stage), showed that radiologic tumour size, pathological stage (AJCC, 8th edition) and KRAS G12D mutation in ctDNA were significantly independent prognostic factors for RFS and OS (Table 3).

**Table 2:** Univariate analysis of clinicopathological variables associated with recurrence-free survival and overall survival in discovery cohort.

Variable	Recurrence-free survival		Overall survival	
	HR (95% CI)	*P*	HR (95% CI)	*P*
*Sex*				
Female	1.0 (reference)	0.195	1.0 (reference)	0.117
Male	1.3 (0.9–1.9)		1.4 (0.9–2.1)	
*Age at surgery, y*				
<70	1.0 (reference)	0.634	1.0 (reference)	0.974
≥70	1.1 (0.7–1.8)		1.0 (0.6–1.6)	
*Smoking history*				
No	1.0 (reference)	0.53	1.0 (reference)	0.311
Yes	1.2 (0.7–2.2)		1.4 (0.7–2.5)	
*Drinking history*				
No	1.0 (reference)		1.0 (reference)	
Yes	1.2 (0.6–2.4)	0.542	1.2 (0.6–2.3)	0.671
*Diabetes mellitus*				
No	1.0 (reference)	0.176	1.0 (reference)	0.194
Yes	0.7 (0.5–1.1)		0.7 (0.5–1.2)	
*First clinical symptom (abdominal pain)*				
No	1.0 (reference)	0.674	1.0 (reference)	0.821
Yes	0.9 (0.6–1.4)		1.0 (0.6–1.4)	
*First clinical symptom (jaundice)*				
No	1.0 (reference)	0.796	1.0 (reference)	0.961
Yes	1.0 (0.6–1.4)		1.0 (0.7–1.5)	
*CEA at diagnosis, ng/mL*				
Normal	1.0 (reference)	0.095	1.0 (reference)	0.081
Elevated	1.4 (0.9–2.2)		1.5 (1.0–2.3)	
*CA19-9 at diagnosis, U/mL*				
Normal	1.0 (reference)	0.102	1.0 (reference)	0.074
Elevated	1.5 (0.9–2.5)		1.7 (1.0–2.9)	
*Pancreatectomy type*				
Distal pancreatectomy	1.0 (reference)	0.947	1.0 (reference)	0.833
Pancreaticoduodenectomy	1.0 (0.7–1.5)		1.0 (0.7–1.6)	
*Radiologic tumour size, cm*	ALL	0.049	ALL	0.026
≤2	1.0 (reference)		1.0 (reference)	
>2, ≤4	1.7 (1.1–2.8)	0.029	1.9 (1.1–3.2)	0.018
>4	2.0 (1.1–3.7)	0.031	2.2 (1.2–4.5)	0.012
*Differentiation degree*				
Poor	1.0 (reference)	0.845	1.0 (reference)	0.267
Medium/well	1.0 (0.6–1.5)		0.8 (0.5–1.2)	
*Perineural invasion*				
No (%)	1.0 (reference)	0.901	1.0 (reference)	0.731
Yes (%)	1.0 (0.6–1.6)		1.1 (0.7–1.8)	
*Pathological margin status*				
R0 (%)	1.0 (reference)	0.091	1.0 (reference)	0.099
R1 (%)	1.5 (0.9–2.3)		1.5 (0.9–2.4)	
*Nodal involvement*				
0 (%)	1.0 (reference)	<0.001	1.0 (reference)	<0.001
≥1, ≤3 (%)	2.2 (1.5–3.3)		2.5 (1.6–3.9)	
≥4 (%)	9.1 (3.6–23.0)		15.2 (5.7–40.3)	
*Pathological stage (AJCC, 8th edition)*	ALL	<0.001	ALL	<0.001
IA	1.0 (reference)		1.0 (reference)	
IB	1.6 (0.8–3.2)	0.15	2.1 (0.9–4.6)	0.069
IIA	2.1 (1.0–4.6)	0.063	3.0 (1.2–7.2)	0.016
IIB	3.4 (1.8–6.5)	<0.001	5.0 (2.3–10.8)	<0.001
III	15.8 (5.7–43.9)	<0.001	31.2 (10.0–98.0)	<0.001
*Adjuvant chemotherapy*				
No	1.0 (reference)	0.083	1.0 (reference)	0.259
Yes	2.4 (0.9–6.6)		1.8 (0.7–4.9)	
*KRAS mutation in ctDNA*				
No	1.0 (reference)	0.001	1.0 (reference)	0.002
Yes	2.2 (1.4–3.4)		2.1 (1.3–3.5)	
*KRAS G12D mutation in ctDNA*				
No	1.0 (reference)	<0.001	1.0 (reference)	<0.001
Yes	4.6 (2.5–8.6)		3.5 (1.9–6.5)	

*CEA* carcinoembryonic antigen, *CA19-9* carbohydrate antigen 19-9, *AJCC* American Joint Committee on Cancer, *ctDNA* cell-free circulating tumour DNA

**Table 3:** Multivariate analysis of clinicopathological variables in relation to recurrence-free survival and overall survival in discovery cohort.

Variable	Recurrence-free survival		Overall survival	
	HR (95% CI)	*P*	HR (95% CI)	*P*
*KRAS mutation in ctDNA*				
No	1.0 (reference)	0.559	1.0 (reference)	0.609
Yes	1.2 (0.6–2.3)		1.2 (0.6–2.3)	
*KRAS G12D mutation in ctDNA*				
No	1.0 (reference)	<0.001	1.0 (reference)	0.001
Yes	5.1 (2.2–11.8)		4.0 (1.8–9.4)	
*Radiologic tumour size, cm*	ALL	0.002	ALL	0.004
≤2	1.0 (reference)		1.0 (reference)	
>2, ≤4	2.3 (1.1–4.9)	0.024	2.1 (1.0–4.5)	0.052
>4	8.4 (2.6–26.9)	<0.001	7.2 (2.2–22.9)	0.001
*Pathological stage (AJCC, 8th edition)*	ALL	<0.001	ALL	<0.001
IA	1.0 (reference)		1.0 (reference)	
IB	0.8 (0.3–2.0)	0.581	1.0 (0.3–3.0)	0.989
IIA	0.3 (0.1–1.2)	0.097	0.5 (0.1–2.1)	0.327
IIB	1.9 (0.8–4.6)	0.16	2.8 (1.1–7.6)	0.04
III	18.4 (6.1–55.7)	<0.001	34.8 (10.1–120.2)	<0.001

*ctDNA* cell-free circulating tumour DNA, *AJCC* American Joint Committee on Cancer

#### Plasma KRAS G12D mutation closely associated with preoperative potential micrometastasis

Survival analysis suggested that patients with KRAS G12D mutation in ctDNA showed significant trend of early recurrence, the median RFS of whom was only 6 months (Fig. 2d). Then a correlation analysis between plasma KRAS G12D mutation and recurrence types was performed, and found that patients with KRAS G12D mutation in ctDNA were more inclined to develop distant metastasis, while those without detectable plasma KRAS G12D mutation had a higher proportion of local recurrence (Table 4, Fig. 2e). The quick appearance of distant metastasis after radical surgery strongly indicated the presence of preoperative potential micrometastasis in patients with plasma KRAS G12D mutation. These results highly supported that plasma KRAS G12D mutation well reflected the systematic tumour burden in resectable PDAC, and ≥0.1% AF of KRAS G12D mutation in ctDNA was able to serve as a promising biomarker of preoperative potential micrometastasis.

**Table 4:** Clinicopathological characteristics of PDAC patients with and without ctDNA KRAS G12D mutations in discover cohort.

Variable	ctDNA KRAS G12D mutation		*P*
	Negative (*N* = 100)	Positive (*N* = 13)	
*Sex*			
Female (%)	38 (86.4%)	6 (13.6%)	0.571
Male (%)	62 (89.9%)	7 (10.1%)	
*Age at surgery, y*			
<70 (%)	78 (87.6%)	11 (12.4%)	0.732
≥70 (%)	22 (91.7%)	2 (8.3%)	
*Smoking history*			
No (%)	88 (88.0%)	12 (12.0%)	1
Yes (%)	12 (92.3%)	1 (7.7%)	
*Drinking history*			
No (%)	90 (87.4%)	13 (12.6%)	0.602
Yes (%)	10 (100.0%)	0 (0.0%)	
*Diabetes mellitus*			
No (%)	73 (91.2%)	7 (8.8%)	0.153
Yes (%)	27 (81.8%)	6 (18.2%)	
*First clinical symptom*			
Abdominal pain			0.692
No (%)	48 (87.3%)	7 (12.7%)	
Yes (%)	52 (89.7%)	6 (10.3%)	
*First clinical symptom*			
Jaundice			0.883
No (%)	56 (88.9%)	7 (11.1%)	
Yes (%)	44 (88.0%)	6 (12.0%)	
*CEA at diagnosis*			
Normal (%)	70 (86.4%)	11 (13.6%)	0.344
Elevated (%)	30 (93.7%)	2 (6.3%)	
*CA19-9 at diagnosis*			
Normal (%)	21 (95.5%)	1 (4.5%)	0.457
Elevated (%)	79 (86.8%)	12 (13.2%)	
*Pancreatectomy type*			
Distal pancreatectomy (%)	31 (86.1%)	5 (13.9%)	0.587
Pancreaticoduodenectomy (%)	69 (89.6%)	8 (10.4%)	
*Radiologic tumour size, cm*			
≤2 (%)	25 (92.6%)	2 (7.4%)	0.374
>2, ≤4 (%)	59 (88.1%)	8 (11.9%)	
>4 (%)	16 (84.2%)	3 (15.8%)	
*Differentiation degree*			
Poor (%)	31 (86.1%)	5 (13.9%)	0.587
Medium/well (%)	69 (89.6%)	8 (10.4%)	
*Perineural invasion*			
No (%)	18 (78.3%)	5 (21.7%)	0.085
Yes (%)	82 (91.1%)	8 (8.9%)	
*Pathological stage (AJCC, 8th edition)*			
IA (%)	14 (93.3%)	1 (6.7%)	0.661
IB (%)	30 (88.2%)	4 (11.8%)	
IIA (%)	12 (85.7%)	2 (14.3%)	
IIB (%)	38 (88.4%)	5 (11,6%)	
III (%)	6 (85.7%)	1 (14.3%)	
*Adjuvant chemotherapy*			
No (%)	6 (100.0%)	0 (0.0%)	1
Yes (%)	94 (87.9%)	13 (12.1%)	
*Recurrent disease*			
No (%)	9 (100.0%)	0 (0.0%)	0.595
Yes (%)	91 (87.5%)	13 (12.5%)	
*Recurrence types*			
Local (%)	36 (97.3%)	1 (2.7%)	0.018
Distant (%)	46 (79.3%)	12 (20.7%)	
Both (%)	9 (100.0%)	0 (0.0%)	

*PDAC* pancreatic ductal adenocarcinoma, *ctDNA* cell-free circulating tumour DNA, *CEA* carcinoembryonic antigen, *CA19-9* carbohydrate antigen 19-9, *AJCC* American Joint Committee on Cancer

### Validation cohort

#### Clinicopathological characteristics of patients

According to the same standard with the discovery cohort, the clinical data of 47 consecutive patients from the Changhai prospective database during August 2016 and December 2016 were collected (Fig. 1). After excluding two patients with intraoperative metastasis and one patient died due to surgical complications within 1 month after surgery, forty-four patients formed the validation cohort. As shown in Table 1, the validation cohort had similar distribution to that of the discovery cohort in most of the clinicopathological variables, but there were no stage III patients in the validation cohort. Median follow-up of the validation cohort was 24.4 months.

#### Verification of the association between plasma KRAS G12D and patient outcomes

**Fig. 3:**
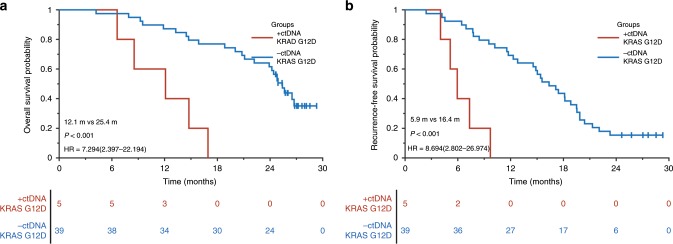
Survival analysis in resectable PDAC patients with or without plasma KRAS G12D mutation in the validation cohort. **a** OS for resectable PDAC patients with (*n* = 5, red) and without (*n* = 39, blue) KRAS G12D mutant in ctDNA. **b** RFS for resectable PDAC patients with (*n* = 5, red) and without (*n* = 39, blue) KRAS G12D mutation in ctDNA.

The association between plasma KRAS G12D and patient outcomes from the discovery cohort was tested in the validation cohort. We performed the same Firefly NGS for ctDNA detection to patients of the validation cohort. Among the 44 patients enrolled, 15 genes in the pancreatic research panel have been detected with an overall detection rate of 34.09% (15/44). These results were similar to the discovery cohort, where only plasma KRAS mutations had a detection rate of >5% (Table 1). Of the nine patients, five patients with KRAS mutations in ctDNA were detected as KRAS G12D subtype. Survival analysis showed that patients with plasma KRAS G12D mutation had significantly poorer prognosis than those without KRAS G12D mutation in ctDNA (Fig. 3a, b), which was consistent with the results of the discovery cohort. The median DFS of patients with plasma KRAS G12D mutation was merely 6 months, which highly suggested that an upfront surgery might not be suitable for such patients even though diagnosed as resectable PDAC.

## Discussion

As a component of liquid biopsy, ctDNA has the most potential clinical application value due to its non-invasive, real-time and highly specific genomic result.^26,27^ Currently, the clinical utility of ctDNA is only approved in metastatic non-small-cell lung cancer (NSCLC) by FDA as a companion diagnostic,^28,29^ while studies on many other cancers have also shown good prospects.^30–36^ The fact that even little variability in process and analysis will result in completely different results restrains the further clinical utility of ctDNA.^37^ In order to solve these problems, some projects, such as the European CANCER-ID, European Liquid Biopsy Academy (ELBA) and European Liquid Biopsy Society (ELBS) networks or the US-based BloodPAC, have been initiated.^38^ In this study, we tried to use a detection method of high specificity to address whether ctDNA in patients with resectable PDAC could possibly serve as a decisive factor for neoadjuvant therapy.

The most popular methods such as ddPCR and NGS-based techniques have their own advantages and disadvantages, separately. ddPCR is associated with high analytical sensitivity down to single molecules and has lower turnaround time than NGS-based approaches when testing single mutations only,^39,40^ but the main disadvantages included sequential testing of mutations and detection of previously unknown mutations is impossible.^18^ In contrast, NGS-targeted sequencing method utilises enrichment of recurrently altered loci by PCR amplification or hybrid capture, allowing de novo identification of molecular alterations, but this inevitably introduces random errors in the amplification process.^20^ In our research, we used the ‘firefly’ method, circularised library and linear amplification to maximise the accuracy and specificity, as the false positive in clinical practice renders the patients to lose the chance to be cured by radical resection.

Different ctDNA concentrations reflect different stages of tumour development and progression (metastatic vs non-metastatic recurrence).^41^ cfDNA in plasma is highly fragmented DNA that is mainly derived from the apoptotic cells, perhaps predominantly from the apoptotic leukocytes (e.g. neutrophils).^42^ During the early stages of cancer, the total amount of ctDNA is usually <0.01% of the total cfDNA, while >10% concentration of ctDNA is believed to be in the clinical metastatic stages, which can be easily detected by imaging examination or tumour marker testing.^18^ The concentration of cfDNA between 0.1% and 10% is thought to be related to micrometastasis of primary tumours, which cannot be undetectable by conventional techniques and critical for surgical outcome and long-time survival.^18^ Therefore, based on Firefly NGS platform, we choose 0.1% as the threshold for ctDNA positive. The combination of a reasonable cut-off value and an accurate detection method with high specificity might be helpful for the stratification of resectable PDAC patients and further provides a proper individualised treatment.

Driver gene mutations represent both primary and metastatic genetic variation during the process of tumour progression. It has been proved that identical mutations in known driver genes, especially KRAS, were present in every metastatic lesion in all PDAC patients, and the genetic similarity among the cells of metastases was higher than that expected for any two random cells taken from a normal tissue.^43^ Moreover, several studies have reported that the correlation of ctDNA with tumour stage, metastatic burden and increased ctDNA mutant allele frequency was observed at the time of recurrence or progression.^44–46^ So, detection of credible tumour-driving gene mutations from ctDNA during the early-stage PDAC might reflect the formation of micrometastasis. In this study, we sequenced the whole exons of 50 genes, including KRAS, P53, SMAD4, CDKN2A and so on. However, only KRAS mutations were identified in 20–25% of patients with early-stage PDAC, while the mutation detection rates of the remaining genes, including driver genes,^47,48^ were significantly lower than that of KRAS, <5%. There might be two reasons: first, the alteration rates of these genes in PDAC tissue are correspondingly lower than KRAS;^22,49^ second, mutation type of suppressor genes like SMAD4 is usually deletion, which makes it difficult to detect in ctDNA.^22,49^ Survival analysis of the discovery cohort demonstrated that KRAS mutation, especially KRAS G12D mutation, in ctDNA was strongly correlated with poor OS and RFS, and this has been proven in tissue also.^50^ Further recurrence analysis showed that patients with plasma KRAS G12D mutation were more likely to develop distant metastasis, especially liver metastasis, in 6 months after surgery, and this highly suggested the existence of micrometastasis before surgery. Importantly, we found similar poor outcome in the validation cohort. Only a 6-month RFS strongly indicated that these patients should receive neoadjuvant therapy rather than upfront surgery, despite resectable PDAC in radiology.

Patients who did not harbour KRAS mutations in ctDNA but had a KRAS mutation in tissue accounted for 74.7% (74/99) (Supplementary Table 5), which indicated a little low sensitivity of 25.3% (25/99) compared with previous studies (30.0%–49.0%).^16,17^ However, this is due to the impairment caused by the threshold of 0.1% AF, which we chose to avoid situations, like ageing, benign tumours and preneoplastic lesions, and better reflect the systematic tumour burden of the patients.^18^ In fact, the Firefly NGS technique allowed a detection of 0.02% mutated DNA,^21^ which suggested that negative results in this study did not mean no detectable ctDNA in preoperative plasma. By maximising the specificity of our method, we could identify the resectable PDAC patients who are most suitable for neoadjuvant therapy instead of upfront surgery. It is more practical in clinical work due to the slim chance of making resectable PDAC patients lose the opportunity of radical surgery.

However, our study still has limitations. Firstly, we performed a retrospective study, in which the PDAC patients were enrolled from a single institution. The conclusions we made from this research still need to be verified in a prospective study. Secondly, the panel we used in this study was established based on a research about the mutational landscape of pancreatic cancer. Genes like GNAS, which was reported to be closely related to the development of pancreatic cyst^51^ and intraductal papillary mucinous neoplasm (IPMN)-associated invasive adenocarcinoma^52^ and might serve as an alternative oncogene in KRAS wild-type PDAC,^49^ were not included and needed to be paid more attention in further study.

In summary, a study screening ctDNA by Firefly NGS in resectable PDAC was performed, and the results showed that KRAS mutations, especially KRAS G12D mutation, in preoperative ctDNA were significantly associated with the outcomes of resectable PDAC. The presence of plasma KRAS G12D mutation showed a strong correlation with early distant metastasis, indicating that these patients had a high probability of preoperative micrometastasis and should possibly receive neoadjuvant therapy first instead of undergoing an upfront surgery. The preoperative detection of plasma KRAS G12D mutation will help in optimising surgical selection of resectable PDAC patients and improving survival time.

## Supplementary information

Supplementary material

## Data Availability

All data in this study are available upon request.
