# Supplementary material for: Preoperative detection of KRAS G12D mutation in ctDNA is a powerful predictor for early recurrence of resectable PDAC patients
Source: Br J Cancer. 2020 Jan 23;122(6):857–67. doi: 10.1038/s41416-019-0704-2 (PMC7078253; doi:10.1038/s41416-019-0704-2)
Supplement: Supplementary file 1 — Supplementary material [file 41416_2019_704_MOESM1_ESM.docx]

**Supplementary Table 1. Pancreatic cancer research panel gene list**

| 1 | KRAS | 11 | PREX2 | 21 | SLIT2 | 31 | MED12 | 41 | FGFR2 |
| --- | --- | --- | --- | --- | --- | --- | --- | --- | --- |
| 2 | TP53 | 12 | KDM6A | 22 | BRCA2 | 32 | MYC | 42 | EPCAM |
| 3 | CDKN2A | 13 | ARID1A | 23 | BRCA1 | 33 | GATA6 | 43 | FANCC |
| 4 | SMAD4 | 14 | ARID1B | 24 | PALB2 | 34 | CDK6 | 44 | MEN1 |
| 5 | MAP2K4 | 15 | PBRM1 | 25 | RPA1 | 35 | NOV | 45 | MSH6 |
| 6 | TGFBR2 | 16 | SMARCA2 | 26 | ATM | 36 | MET | 46 | PALLD |
| 7 | ACVR1B | 17 | SMARCA4 | 27 | STK11 | 37 | SOX9 | 47 | PMS2 |
| 8 | RNF43 | 18 | MLL2 | 28 | MLH1 | 38 | ERBB2 | 48 | APC |
| 9 | SF3B1 | 19 | ROBO1 | 29 | MSH2 | 39 | PIK3CA | 49 | VHL |
| 10 | BRAF | 20 | ROBO2 | 30 | PTPRF | 40 | PIK3R3 | 50 | RBM10 |

**Supplementary Table 2. Details of KRAS mutation profiles in plasma by Firefly NGS and ddPCR.**

| **Number** | **Firefly NGS** | | **ddPCR** | |
| --- | --- | --- | --- | --- |
|  | **Type** | **AF** | **Type** | **AF** |
| NO.2 | KRAS.G12V | 10.23% | KRAS.G12V | 11.78% |
| NO.4 | KRAS.G12D | 1.30% | KRAS.G12D | 1.07% |
| NO.5 | KRAS.G12R | 0.49% | KRAS.G12R | 0.47% |
| NO.6 | KRAS.G12D | 0.63% | KRAS.G12D | 1.04% |
| NO.7 | KRAS.G12V | 38.20% | KRAS.G12V | 34.50% |
| NO.9 | KRAS.G12D | 2.61% | KRAS.G12D | 1.77% |
| NO.10 | KRAS.G12V | 0.50% | KRAS.G12V | 0.36% |
| NO.11 | KRAS.G12R | 1.88% | KRAS.G12R | 1.56% |
| NO.12 | KRAS.G12D | 5.39% | KRAS.G12D | 7.07% |
| NO.13 | KRAS.G12C | 1.24% | KRAS.G12C | 1.53% |
| NO.15 | KRAS.G12D | 29.28% | KRAS.G12D | 25.20% |
| NO.19 | KRAS.G12D | 0.33% | KRAS.G12D | 0.67% |
| NO.24 | KRAS.Q61H | 4.05% | None | 0% |
| NO.26 | KRAS.G12V | 0.60% | KRAS.G12V | 0.47% |
| NO.27 | KRAS.G12D | 18.23% | KRAS.G12D | 16.19% |
| NO.28 | KRAS.G12V | 1.56% | KRAS.G12V | 2.72% |
| NO.29 | KRAS.G12D | 0.25% | KRAS.G12D | 0.50% |
| NO.30 | KRAS.G12D | 7.20% | KRAS.G12D | 8.01% |
| NO.31 | KRAS.G12D | 0.32% | KRAS.G12D | 0.35% |
| NO.32 | KRAS.G12D | 2.49% | KRAS.G12D | 2.28% |
| NO.33 | KRAS.G13D | 1.67% | None | 0% |
| NO.34 | KRAS.G12V | 0.48% | KRAS.G12V | 0.93% |
| NO.36 | KRAS.Q61R | 0.38% | None | 0% |
| NO.37 | KRAS.G12D | 9.54% | KRAS.G12D | 8.95% |
| NO.38 | KRAS.G12V | 2.53% | KRAS.G12V | 1.33% |
| NO.39 | KRAS.G12D | 12.25% | KRAS.G12D | 15.40% |

NGS，next generation sequencing; ddPCR, droplet digital PCR; AF, allele frequency.

**Supplementary Table 3. Details of KRAS mutation profiles in plasma by Firefly NGS and in tissue by NGS.**

| **Number** | **Firefly NGS in plasma** | | **NGS in tissue** | |
| --- | --- | --- | --- | --- |
|  | **Type** | **AF** | **Type** | **AF** |
| NO.2 | KRAS.G12V | 10.23% | KRAS.G12V | 15.30% |
| NO.4 | KRAS.G12D | 1.30% | KRAS.G12D | 68.46% |
| NO.5 | KRAS.G12R | 0.49% | KRAS.G12R | 13.90% |
| NO.6 | KRAS.G12D | 0.63% | KRAS.G12D | 11.81% |
| NO.7 | KRAS.G12V | 38.20% | KRAS.G12V | 30.60% |
| NO.9 | KRAS.G12D | 2.61% | None | 0% |
| NO.10 | KRAS.G12V | 0.50% | KRAS.G12V | 25.22% |
| NO.11 | KRAS.G12R | 1.88% | KRAS.G12R | 14.39% |
| NO.12 | KRAS.G12D | 5.39% | KRAS.G12D | 16.40% |
| NO.13 | KRAS.G12C | 1.24% | KRAS.G12C | 25.70% |
| NO.15 | KRAS.G12D | 29.28% | KRAS.G12D | 43.69% |
| NO.19 | KRAS.G12D | 0.33% | KRAS.G12D | 18.99% |
| NO.24 | KRAS.Q61H | 4.05% | KRAS.Q61H | 31.00% |
| NO.26 | KRAS.G12V | 0.60% | KRAS.G12V | 21.22% |
| NO.27 | KRAS.G12D | 18.23% | KRAS.G12D | 15.39% |
| NO.28 | KRAS.G12V | 1.56% | KRAS.G12V | 17.00% |
| NO.29 | KRAS.G12D | 0.25% | KRAS.G12D | 22.90% |
| NO.30 | KRAS.G12D | 7.20% | KRAS.G12D | 32.70% |
| NO.31 | KRAS.G12D | 0.32% | KRAS.G12D | 5.20% |
| NO.32 | KRAS.G12D | 2.49% | KRAS.G12D | 7.60% |
| NO.33 | KRAS.G13D | 1.67% | KRAS.G13D | 21.30% |
| NO.34 | KRAS.G12V | 0.48% | KRAS.G12V | 11.10% |
| NO.36 | KRAS.Q61R | 0.38% | KRAS.Q61R | 20.29% |
| NO.37 | KRAS.G12D | 9.54% | KRAS.G12D | 1.50% |
| NO.38 | KRAS.G12V | 2.53% | KRAS.G12V | 1.40% |
| NO.39 | KRAS.G12D | 12.25% | KRAS.G12D | 5.30% |

NGS，next generation sequencing; AF, allele frequency.

**Supplementary table 4.** **Clinicopathological characteristics of PDAC patients with and without ctDNA KRAS mutations in discover cohort.**

| **Variable** | **ctDNA KRAS mutation** | | **P** |
| --- | --- | --- | --- |
|  | **Negative (N=87)** | **Positive (N=26)** |  |
| Sex  Female (%)  Male (%) | 34(77.3%)  53(76.8%) | 10(22.7%)  16(23.2%) | 1.000 |
| Age at surgery, y  < 70 (%)  ≥ 70 (%) | 67(75.3%)  20(83.3%) | 22(24.7%)  4(16.7%) | 0.586 |
| Smoking history  No (%)  Yes (%) | 79(79.0%)  8(61.5%) | 21(21.0%)  5(38.5%) | 0.172 |
| Drinking history  No (%)  Yes (%) | 81(78.6%)  6(60.0%) | 22(21.4%)  4(40.0%) | 0.235 |
| Diabetes mellitus  No (%)  Yes (%) | 62(77.5%)  25(75.8%) | 18(22.5%)  8(24.2%) | 0.811 |
| First clinical symptom  Abdominal pain  No (%)  Yes (%) | 42(76.4%)  45(77.6%) | 13(23.6%)  13(22.4%) | 1.000 |
| First clinical symptom  Jaundice  No (%)  Yes (%) | 48(76.2%)  39(78.0%) | 15(23.8%)  11(22.0%) | 1.000 |
| CEA at diagnosis  Normal (%)  Elevated (%) | 62(76.5%)  25(78.1%) | 19(23.5%)  7(21.9%) | 1.000 |
| CA19-9 at diagnosis  Normal (%)  Elevated (%) | 18(81.8%)  69(75.8%) | 4(18.2%)  22(24.2%) | 0.778 |
| Pancreatectomy type  Distal pancreatectomy (%)  Pancreaticoduodenectomy (%) | 29(80.6%)  58(75.3%) | 7(19.4%)  19(24.7%) | 0.636 |
| Radiologic tumor size, cm  ≤2 (%)  ＞2, ≤4 (%)  ＞4 (%) | 23(85.2%)  50(74.6%)  14(73.7%) | 4(14.8%)  17(25.4%)  5(26.3%) | 0.520 |

**Supplementary table 4. (Continued)**

| **Variable** | **ctDNA KRAS mutation** | | **P** |
| --- | --- | --- | --- |
|  | **Negative (N=87)** | **Positive (N=26)** |  |
| Differentiation degree  Poor (%)  Medium/Well (%) | 26(72.2%)  61(79.2%) | 10(27.8%)  16(20.8%) | 0.474 |
| Perineural invasion  No (%)  Yes (%) | 15(65.2%)  72(80.0%) | 8(34.8%)  18(20.0%) | 0.166 |
| Pathological margin status  R0 (%)  R1 (%) | 69(78.4%)  18(72.0%) | 19(21.6%)  7(28.0%) | 0.502 |
| Nodal involvement  0 (%)  ≥1，≤3 (%)  ≥4 (%) | 51(79.7%)  32(74.4%)  4(66.7%) | 13(20.3%)  11(25.6%)  2(33.3%) | 0.382 |
| Pathological stage (AJCC, 8th edition)  IA (%)  IB (%)  IIA (%)  IIB (%)  III (%) | 13(86.7%)  26(76.5%)  11(78.6%)  32(74.4%)  5(71.4%) | 2(13.3%)  8(23.5%)  3(21.4%)  11(25,6%)  2(28.6%) | 0.395 |
| Adjuvant chemotherapy  No (%)  Yes (%) | 5(83.3%)  82(76.6%) | 1(16.7%)  25(23.4%) | 1.000 |
| Recurrent disease  No (%)  Yes (%) | 8(88.9%)  79(76.0%) | 1(11.1%)  25(24.0%) | 0.682 |
| Recurrence types  Local (%)  Distant (%)  Both (%) | 31(83.8%)  42(72.4%)  6(66.7%) | 6(16.2%)  16(27.6%)  3(33.3%) | 0.406 |

PDAC, pancreatic ductal adenocarcinoma; ctDNA, cell-free circulating tumor DNA; CEA, carcinoembryonic antigen; CA19-9, carbohydrate antigen 19-9; AJCC, American Joint Committee on Cancer.

**Supplementary table 5. KRAS mutation detected in tissue by NGS and in ctDNA by Firefly NGS of PDAC patients in discover cohort.**

|  | Tissue KRAS mutation | | Tissue KRAS wild type | | Total |
| --- | --- | --- | --- | --- | --- |
| ctDNA KRAS positive | 25 | 1 | | 26 | |
| ctDNA KRAS negative | 74 | 13 | | 87 | |
| Total | 99 | 14 | | 113 | |

**Figure S1.** **Representative image of cresyl violet stain on frozen resection of PDAC sample.** The frozen resection was stained by cresyl violet to distinguish tumor cell and stroma before assessment. The representative image below indicated a tumor cellularity of around 40% (red arrow: tumor cell, yellow arrow: stroma). The scale bar was shown in the image.


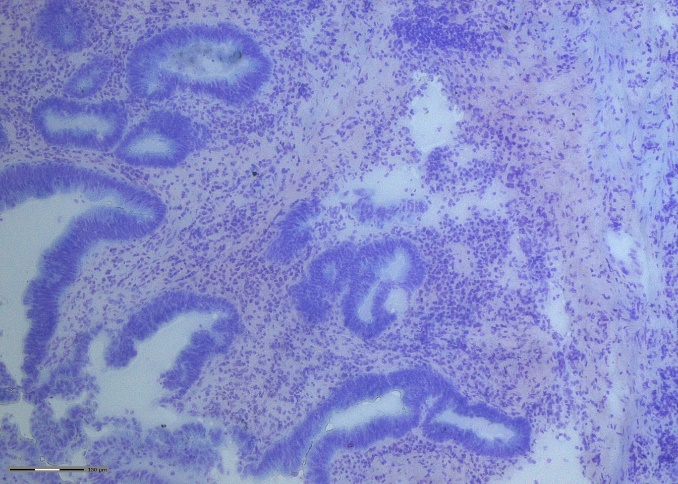


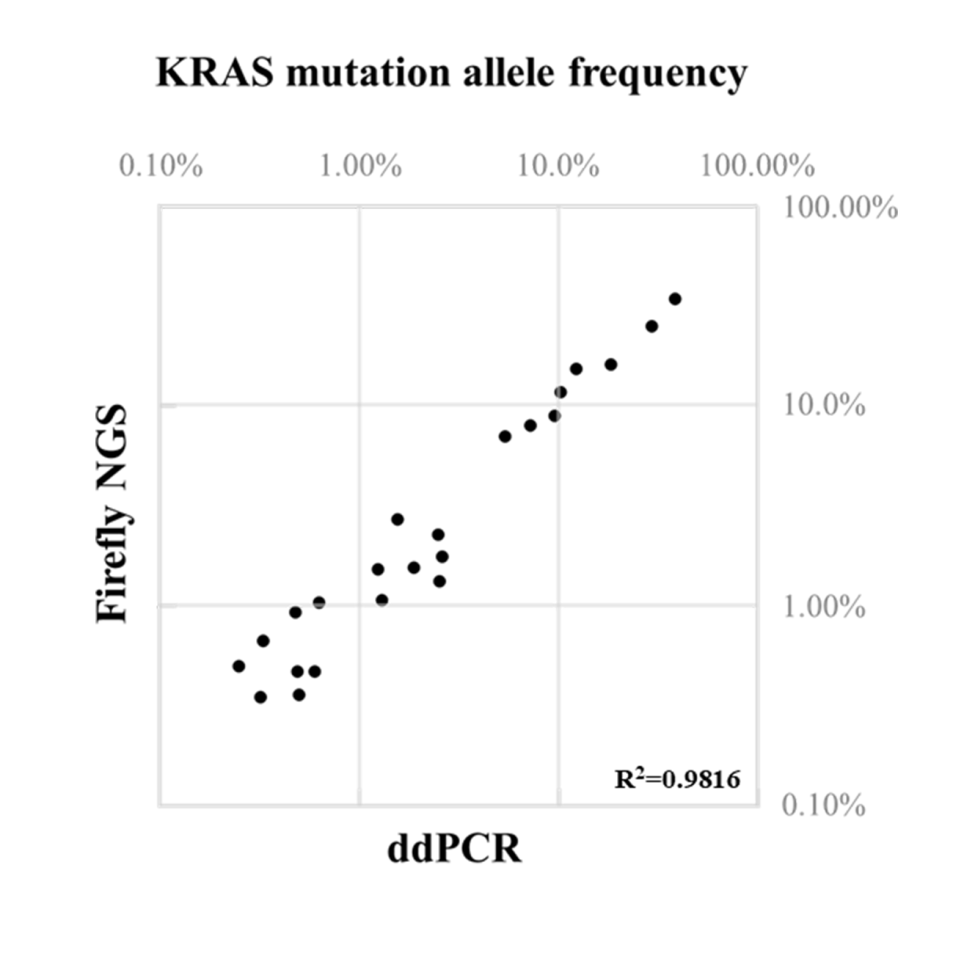
**Figure S2. Correlation analysis of plasma KRAS mutation allele frequency by Firefly-NGS (≥0.1%) and ddPCR.** Samples with plasma KRAS mutation allele frequency ≥0.1% by Firefly-NGS was detected again using ddPCR (KRAS G12D/G12V/G12R/G12C/G12A/G12S). Correlation analysis showed high consistency in the AF results between these two methods (R^2^=0.98).


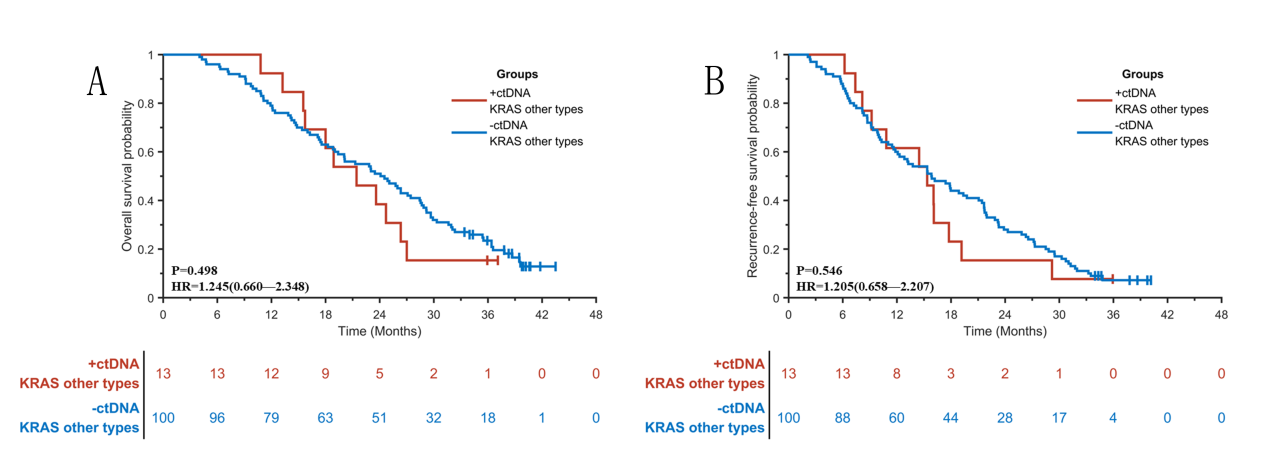
**Figure S3. Survival analysis in resectable PDAC patients with or without plasma KRAS other mutations in discovery cohort.** (A) OS for resectable PDAC patients with (n=13, red) and without (n=100, blue) KRAS other mutants in ctDNA. (B) RFS for resectable PDAC patients with (n=13, red) and without (n=100, blue) KRAS other mutants in ctDNA.
